# Keratosis pilaris and filaggrin loss‐of‐function mutations in patients with atopic dermatitis – Results of a Finnish cross‐sectional study

**DOI:** 10.1111/1346-8138.16477

**Published:** 2022-05-26

**Authors:** Alexander Salava, Ville Salo, Anita Remitz

**Affiliations:** ^1^ Helsinki University Hospital, Skin and Allergy Hospital Helsinki Finland

**Keywords:** atopic dermatitis, filaggrin, keratosis pilaris, mutation, observational study

## Abstract

Keratosis pilaris (KP) associates with epidermal barrier defects in atopic dermatitis (AD) but its role in disease severity and concomitant atopic diseases seems to vary between populations. We performed a cross‐sectional observational study with 502 randomly selected AD patients of a Finnish tertiary health care center. At a single clinical examination, disease severity (Rajka Langeland severity score and EASI), clinical signs and patient history were evaluated and total IgE levels and frequent filaggrin (FLG) loss‐of‐function mutations were investigated. There was no link with disease severity (*p* = 0.649, 95% CI 0.569–0.654), asthma (*p* = 0.230, 95% CI 0.206–0.281) or atopic sensitization (*p* = 0.351, 95% CI 0.309–0.392). Keratosis pilaris was significantly associated with palmar hyperlinearity (*p* < 0.000, 95% CI 0.000–0.006, OR 4.664, 95% CI 2.072–10.496) and the filaggrin loss‐of‐function mutation 2282del4 (*p* < 0.000, 95% CI 0.000–0.009, OR 4.917, 95%CI 1.961–12.330). The prevalence of KP in the cohort was generally low and KP seems to be infrequent in Finnish AD patients. This may be explained by the fact that the tested FLG loss‐of‐function mutations are rarer in the Finnish population compared for example, with central Europe or Asia. Mutations in other locations of the FLG gene or other genes of the epidermal barrier may play a more important role.

## 1 INTRODUCTION

Keratosis pilaris (KP) is a common inherited disorder which has been associated with filaggrin (FLG) loss‐of‐function (LoF) mutations, atopic dermatitis (AD), and ichtyosis vulgaris.^1^ FLG mutations are frequent in the European population with an approximate prevalence of 7% and carriers have an increased risk for atopic diseases with a wide clinical spectrum.^2^ Research has shown that associated clinical characteristics and the genetic backgrounds are heterogenous and have differed considerably between different studies and countries.^3^ KP has been shown to associate with epidermal barrier defects in atopic dermatitis but its role in disease severity prediction and concomitant atopic diseases is less certain and seems to vary between populations. We aimed to explore the occurrence of KP in Finnish patients with AD and to investigate the link to frequent FLG LoF mutations.

## 2 METHODS

We performed a cross‐sectional observational study with 502 randomly selected AD patients of a Finnish tertiary health care center. Patient recruitment was carried out in the years 2011–2015 and inclusion criterion was AD. During a single clinical examination, in each patient, disease severity (Rajka Langeland severity score [RLS] and EASI), clinical signs (KP, palmar hyperlinearity) and patient history were evaluated. Data on disease onset, hereditary factors regarding first‐degree relatives, contact allergy (confirmed by patch testing), prick positivity (positivity to any of following: birch, timothy, mug wort, cat, dog, horse, house dust mite and *Cladosporium herbarum*), peanut allergy (confirmed by allergy tests and concomitant symptoms) and atopic comorbidities were based on the patient history. At the clinical visit, we investigated the total serum IgE‐levels and a panel of FLG null‐mutations. Genetic testing was carried out by sequencing four FLG LoF mutations that are frequent in the European population (R501X, 2282del4, R2447X, S3247X), two FLG LoF mutations enriched in the Finnish population (S1020X, V603M), and the 12‐repeat allele (rs12730241). In addition, we sequenced 59 functional variants of 10 different genes associated with skin barrier defects (e.g., filaggrin‐2). The genetic testing, sequencing methods and complete list of genotyped variants have been described in detail in a former study.^4^ All laboratory tests were carried out in the Laboratory of Helsinki University Hospital according to accredited methods. The ethics committee of the University Hospital Helsinki, Finland approved the study protocol.

## 3 RESULTS

**FIGURE 1:**
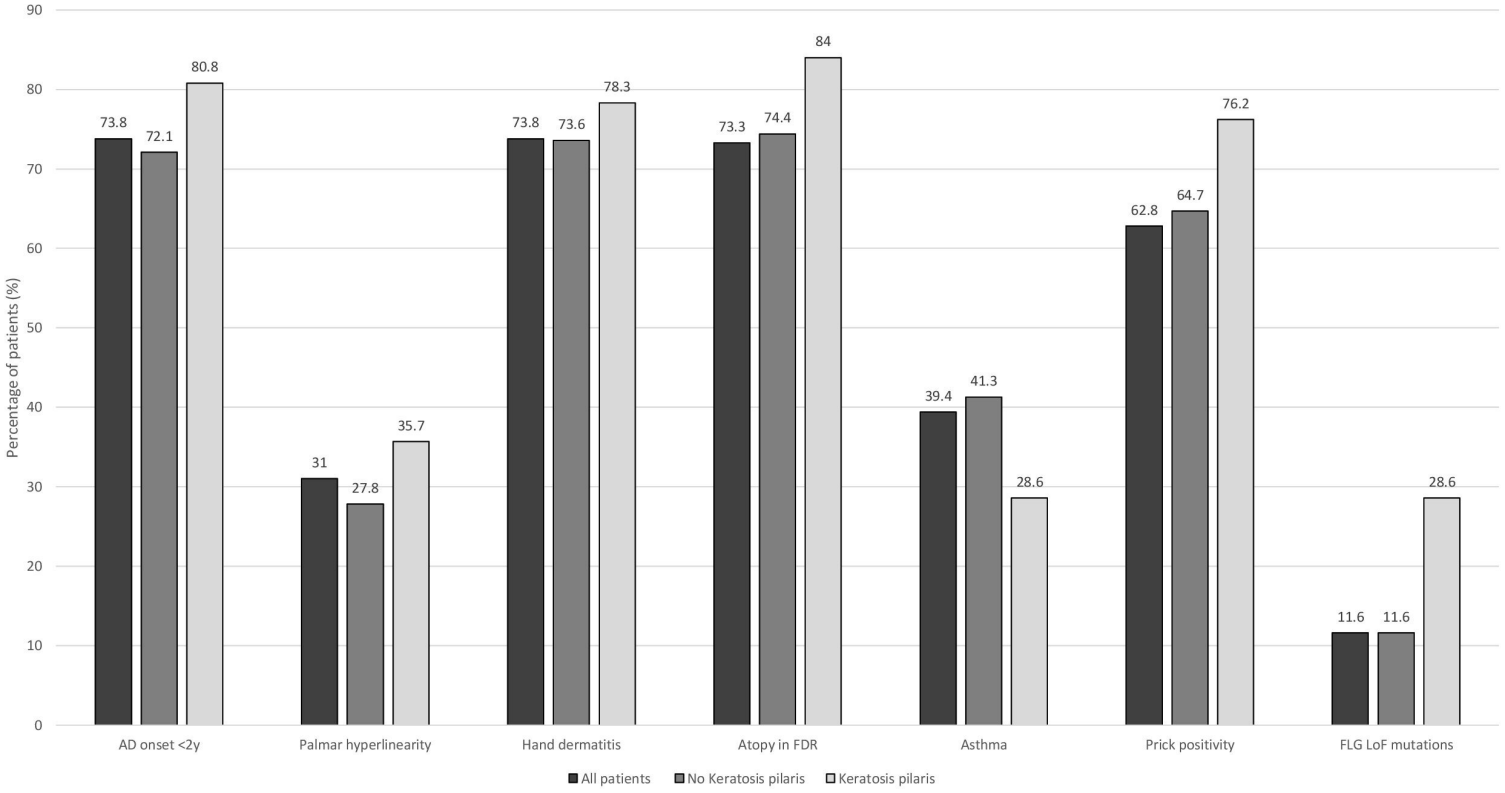
Percentages (%) of patients with different clinical signs, factors in patient history, prick positivity and filaggrin loss‐of‐function mutations. Prick positivity was defined as positive result to any of the following: Birch, timothy, mug wort, cat, dog, horse, house dust mite and *Cladosporium herbarum*. AD, Atopic dermatitis; FDR, first‐degree relative; FLG, filaggrin, LoF, loss‐of‐function

There was a relatively low number of patients with KP in the investigated cohort (28 KP vs. 319 with no KP). KP was not associated with AD severity based on EASI at the clinical visit (*p* = 0.3232, 95% CI 0.276–0.357) and RLS (*p* = 0.649, 95% CI 0.569–0.654), representing severity of the past year. In addition, there were no significant links to total serum IgE levels, early AD onset or positive history of hand dermatitis, contact allergy, asthma, prick positivity to frequent aeroallergens or peanut allergy (Table 1, Figure 1). There were no patients in the cohort with clinically diagnosed ichtyosis vulgaris. Prevalence of dry skin, atopy or ichtyosis vulgaris was not increased in first‐degree relatives of KP patients (Table 2). KP was significantly associated with the FLG LoF mutation 2282del4 (*p* < 0.000, 95% CI 0.000–0.009, OR 4.917, 95% CI 1.961–12.330), but not with the other sequenced mutations R50X, R2447X or combined heterozygosity FLG_152285076_12 & RS61816761_AG or RS138726443_GA & FLG_152285076_12. We found no association of AD with the tested functional variants of epidermal barrier genes. Patients with KP showed a significantly higher occurrence of palmar hyperlinearity (*p* < 0.000, 95% CI 0.000–0.006, OR 4.664, 95% CI 2.072–10.496).

**TABLE 1:** Patient characteristics, AD severity parameters and FLG loss‐of‐function mutations

	All patients	No keratosis pilaris	Keratosis pilaris	Comparison no KP vs KP, *p*‐values (CI)	Comparison no KP vs KP, Odds ratio (CI)
Number of patients, M:F (%)	502 188 (37.5):314 (62.5)	319 (91.9%) 116 (36.4):203 (63.6)	28 (8.1%) 9 (32.1%):19 (67.9%)	0.656^a^ (0.645–0.726)	0.829^a^ (0.363–1.892)
Age at clinical visit (years) median, IQR (mean, range)	28.38, 21.01–40.05 (32.08, 4.79–79.90)	28.36, 20.97–40.28 (31.88, 4.79–73.18)	25.81, 20.60–31.33, (28.51, 15.61–66.34)	0.186^b^ (0.144–0.211)	–
RLS median, IQR (mean, range)	6.00, 4.00–7.63 (5.81, 3.0–9.0)	5.50, 4.00–8.00 (5.80, 3.0–9.0)	5.50 (4.00–7.75), 5.63 (3.0–9.0)	0.649^b^ (0.569–0.654)	–
EASI median, IQR (mean, range)	4.05, 0.90–11.78 (8.24, 0.00–57.60)	4.10, 0.90–12.30 (8.09, 0.00–46.00)	1.80, 0.60–9.40, (5.25, 0.00–17.00)	0.323^b^ (0.276–0.357)	–
Serum total IgE median, IQR (mean, range)	570.50, 158.50–2993.00 (2–189 500)	545.00, 154.50–2702.00 (3512.93, 2.00–74 210)	341.00, 115.00–2470.00, (2954.85, 22.00–27253.00)	0.406^b^ (0.352–0.437)	–
FLG LoF mutation carrier (any), yes, no (%)	58 (11.6), 444 (88.4)	37 (11.6), 282 (88.4)	8 (28.6%), 20 (71.4%)	**0.010** ^a^ (0.006–0.030)	3.049^a^ (1.254–7.414)
R50X, yes, no (%)	9 (1.8), 493 (98.2)	3 (0.9), 316 (99.1)	1 (3.6), 27 (96.4)	0.211^a^ (0.209–0.285)	1.027^a^ (0.956–1.104)
2282del4, yes, no (%)	37 (7.4), 465 (92.6)	24 (7.5), 295 (92.5)	8 (28.6), 20 (71.4)	**0.000** ^a^ (0.000–0.009)	4.917^a^ (1.961–12.330)
R2447X, yes, no (%)	14 (2.8), 488 (97.2)	10 (3.1), 309 (96.9)	0 (0.0)	0.342^a^ (0.283–0.668)	0.917^a^ (0.888–0.947)^c^
Compound heterozygosity 1, yes, no (%)	1 (0.2), 501 (99.8)	0 (0.0)	1 (0.2), 501 (99.8)	0.001^a^ (0.054–0.101)	1.037^a^ (0.966–1.114)^d^
Compound heterozygosity 2, yes, no (%)	1 (0.2), 501 (99.8)	0 (0.0)	0 (0.0)	N/A	N/A

*Note*: Statistical significance was set at *p* < 0.05; indicated in the table as values with bolded font.

Abbreviations: AD, Atopic dermatitis; CI, 95% Confidence interval; Compound heterozygosity 1, FLG_152285076_12 & RS61816761_AG; Compound heterozygosity 2, RS138726443_GA & FLG_152285076_12; EASI, Eczema Area and Severity Index; FLG, Filaggrin; IQR, Interquartile range; KP, Keratosis pilaris; N/A, LoF, loss‐of‐function; Not applicable; RLS, Rajka Langeland severity score.

^a^ Pearson's Chi Square test.

^b^ Mann Whitney *U* test.

^c^ OR for no keratosis pilaris.

^d^ OR for keratosis pilaris.

**TABLE 2:** Clinical signs and data from patients' history

	All patients	No keratosis pilaris	Keratosis pilaris	Comparison^a^ no KP vs KP, *p*‐values (CI)	Comparison no KP vs KP, Odds ratio (CI)
AD onset <2 years, >2 years (%), missing	335 (73.8), 119 (26.2), 48	207 (72.1), 80 (27.9), 32	21 (80.8%), 5 (19.2%)	0.343 (0.338–0.423)	0.616 (0.225–1.690)
Palmar hyperlinearity yes, no (%), missing	111 (31.0), 247 (69.0), 144	88 (27.8), 228 (72.2), 3	18 (64.3), 10 (35.7)	**0.000** (0.000–0.006)	4.664 (2.072–10.496)
Hand dermatitis yes, no (%), missing	340 (73.8), 121 (26.2), 41	218 (73.6), 78 (26.4), 23	18 (78.3%), 5 (21.7%), 5	0.627 (0.598–0.863)	0.941 (0.751–1.180)
Contact allergy yes, no (%), missing)	65 (19.1), 276 (80.9), 161	38 (18.1), 172 (81.9), 109	3 (17.6%), 14 (82.4), 11	0.963 (0.894–1.000)	0.995 (0.791–1.251)
FDR history yes, no (%), missing					
Atopy	340 (73.3), 124 (26.7), 38	224 (74.4), 77 (25.6), 18	21 (84.0%), 4 (16.0%), 3	0.359 (0.317–0.401)	0.886 (0.737–1.064)
Dry skin	259 (71.2), 105 (28.8), 138	166 (72.2), 64 (27.8), 89	12 (70.6%), 5 (29.4%), 11	0.889 (0.794–1.000)	1.022 (0.745–1.404)
Ichtyosis vulgaris	7 (3.4), 197 (96.6), 204	2 (1.6), 126 (98.4), 191	1 (12.5%), 7 (87.5%), 20	0.189 (0.155–0.224)	0.125 (0.13–1.236)
Asthma, yes, no (%), missing	197 (39.4), 303 (60.6), 2	131 (41.3), 186 (58.7), 2	8 (28.6%), 20 (71.4%)	0.230 (0.206–0.281)	0.568 (0.243–1.329)
Prick positivity yes, no, missing	273 (62.8), 162 (37.2), 67	176 (64.7), 96 (35.3), 47	16 (76.2), 5 (23.8), 7	0.351 (0.309–0.392)	0.849 (0.658–1.096)
Peanut prick (positive, negative, missing)	130 (29.8), 206 (70.2), 66	74 (27.1), 199 (72.9), 46	9 (42.9), 12 (57.1%), 7	0.122 (0.118–0.181)	0.632 (0.372–1.075)
Peanut allergy yes, no (%), missing	150 (35.9), 268 (64.1), 84	95 (33.5), 189 (66.5), 35	12 (42.9), 14 (53.8), 2	0.192 (0.155–0.224)	0.725 (0.464–1.133)

*Note*: Prick positivity: positivity to any of following aeroallergens, birch, timothy, mug wort, cat, dog, horse, house dust mite and *Cladosporium herbarum*. Statistical significance was set at *p* < 0.05; indicated in the table as values with bolded font.

Abbreviations: AD, Atopic dermatitis; CI, 95% Confidence interval.; FDR, First degree relative; KP, Keratosis pilaris.

^a^ Pearson's Chi Square test.

## 4 DISCUSSION

The study shows that KP in Finnish AD patients may be associated with the FLG LoF mutation 2282del4 and the clinical sign of palmar hyperlinearity.^5^ There have been observations that link KP with asthma, atopic sensitization, hand dermatitis and contact allergies based on mechanistic studies and increased epicutaneous penetration of allergens.^6^, ^7^, ^8^ We did not observe similar effects in our cohort although the prevalence of atopic comorbidities (e.g., asthma) was relatively high in the studied Finnish AD patients. In addition, there were no indications that KP represents a marker of AD severity or early onset in Finnish patients which has been discussed by some authors.^9^

Limitations of the study were the generally low number of patients with KP and the cross‐sectional design which represents only a time point in the patients' history. In addition, the data was mostly based on retrospective patient‐derived information and there was only a limited panel of FLG mutations tested. We also did not measure blood eosinophil levels or other possibly laboratory values relevant in atopic dermatitis, because we wanted to concentrate on FLG‐mutations and clinical characteristics.

Former studies have underlined the genetic heterogeneity of atopic dermatitis and its association with KP.^3^ FLG LoF mutations are the most important known genetic risk factors for AD with possible disease modifying relevance and predictive value (e.g., early onset, severe disease course, concomitant atopic diseases such as asthma).^6^ However, there are substantial ethnic differences in the types of FLG mutations found in AD.^1^ Based on large amount of sequencing data, R501X and 2282del4 seem to be the major FLG mutations in Europe and FLG P478S and C3321delA variants in Asia, but genetic or clinical data on concomitant KP is very limited.^10^

Although genetic sequencing from different countries have shown links to FLG‐mutations, especially R501X and 2282del4, they do not account for the complete KP phenotype or explain the wide clinical spectrum.^1^, ^2^ For example, clinical studies and genetic sequencing data from Croatia,^11^ the United States,^12^ the United Kingdom,^5^ Austria^13^ and Singapore^14^ have underlined geographical differences regarding FLG‐mutations, comorbidity with atopic dermatitis and associated clinical signs. To our knowledge there are no former studies on clinical KP characteristics or genetic testing in Finnish patients with concomitant AD.

Thus, association of KP with AD seems to be infrequent in Finnish patients which may be explained by the fact that the tested FLG LoF mutations are overall much rarer in the Finnish population compared for example, with central Europe or Asia.^4^ Mutations in other locations of the FLG gene or other genes of the epidermal barrier may play a more important role in Finnish patients and need further research.^15^

## CONFLICT OF INTEREST

The authors state no conflicts of interest.
